# New Multidrug Efflux Inhibitors for Gram-Negative Bacteria

**DOI:** 10.1128/mBio.01340-20

**Published:** 2020-07-14

**Authors:** Robert L. Marshall, Georgina S. Lloyd, Amelia J. Lawler, Sarah J. Element, Jaswant Kaur, Maria Laura Ciusa, Vito Ricci, Andreas Tschumi, Holger Kühne, Luke J. Alderwick, Laura J. V. Piddock

**Affiliations:** a Institute of Microbiology and Infection, College of Medical and Dental Sciences, University of Birmingham, Birmingham, United Kingdom; b Roche Pharma Research and Early Development, Roche Innovation Center Basel, Basel, Switzerland; c School of Biosciences, College of Life and Environmental Sciences, University of Birmingham, Birmingham, United Kingdom; Lahey Hospital and Medical Center

**Keywords:** efflux inhibitors, GFP, high throughput, RamA

## Abstract

Active efflux of antibiotics preventing their accumulation to toxic intracellular concentrations contributes to clinically relevant multidrug resistance. Inhibition of active efflux potentiates antibiotic activity, indicating that efflux inhibitors could be used in combination with antibiotics to reverse drug resistance. Expression of *ramA* by Salmonella enterica serovar Typhimurium increases in response to efflux inhibition, irrespective of the mode of inhibition. We hypothesized that measuring *ramA* promoter activity could act as a reporter of efflux inhibition. A rapid, inexpensive, and high-throughput green fluorescent protein (GFP) screen to identify efflux inhibitors was developed, validated, and implemented. Two chemical compound libraries were screened for compounds that increased GFP production. Fifty of the compounds in the 1,200-compound Prestwick chemical library were identified as potential efflux inhibitors, including the previously characterized efflux inhibitors mefloquine and thioridazine. There were 107 hits from a library of 47,168 proprietary compounds from L. Hoffmann La Roche; 45 were confirmed hits, and a dose response was determined. Dye efflux and accumulation assays showed that 40 Roche and three Prestwick chemical library compounds were efflux inhibitors. Most compounds had specific efflux-inhibitor-antibiotic combinations and/or species-specific synergy in antibiotic disc diffusion and checkerboard assays performed with Escherichia coli, Pseudomonas aeruginosa, Acinetobacter baumannii, and *Salmonella* Typhimurium. These data indicate that both narrow-spectrum and broad-spectrum combinations of efflux inhibitors with antibiotics can be found. Eleven novel efflux inhibitor compounds potentiated antibiotic activities against at least one species of Gram-negative bacteria, and data revealing an E. coli mutant with loss of AcrB function suggested that these are AcrB inhibitors.

## Abstract (precis)

Multidrug-resistant Gram-negative bacteria pose a serious threat to human and animal health. Molecules that inhibit multidrug efflux offer an alternative approach to resolving the challenges caused by antibiotic resistance, by potentiating the activity of old, licensed, and new antibiotics. We have developed, validated, and implemented a high-throughput screen and used it to identify efflux inhibitors from two compound libraries selected for their high chemical and pharmacological diversity. We found that the new high-throughput screen is a valuable tool to identify efflux inhibitors, as evidenced by the 43 new efflux inhibitors described in this study.

## INTRODUCTION

Antibiotic-resistant bacteria pose a serious threat to modern medicine and human life and therefore have been identified by global agencies such as the World Health Organization (WHO) as a major threat to society (https://www.who.int/news-room/fact-sheets/detail/antibiotic-resistance). Only a minority of available antibacterials are active against Gram-negative bacteria. This is particularly true for those species on the WHO list of global priority pathogens for which there is a critical need for new antibiotics (1). This is due to the permeability barrier of the outer membrane preventing access of many drugs to intracellular targets and to the presence of multidrug resistance (MDR) tripartite efflux pumps that confer intrinsic resistance (2). As the outer membrane restricts drug access to the cell and efflux pumps actively remove drugs once they have gained access, there is a natural synergy between these two mechanisms (2, 3). Under laboratory conditions, deletion, mutational inactivation, or inhibition of MDR efflux systems causes increased susceptibility to a wide variety of antibiotics.

Mutations in regulatory genes increase production of efflux pumps such as the AcrAB-TolC resistance-nodulation-division (RND) MDR pump of *Enterobacteriaceae* and its close homologues in other Gram-negative bacteria (4–10). In addition to RND MDR efflux systems conferring drug resistance, altered expression influences the ability of the bacterium to colonize and infect its host and/or to form a biofilm leading to chronic infections (11, 12). Molecules that inhibit efflux offer an alternative approach to resolving the challenges caused by antibiotic resistance, by potentiating the activity of old, licensed, and new antibiotics (11, 12). Such efflux inhibitors may also act as antivirulence or antibiofilm agents, providing secondary effects that may be of clinical benefit.

In *Salmonella*, multiple transcriptional regulators are known to affect expression of the AcrAB-TolC MDR efflux pump, by either repressing or activating the promoter of the efflux pump genes (4, 13, 14). Expression of these transcriptional regulators is altered in response to environmental conditions and cellular activity. In particular, RamA, a transcriptional activator that increases expression of *acrAB*, is sensitive to efflux inhibition irrespective of the method of inhibition: deletion of an efflux pump gene, chemical inhibition, or dissipation of the proton motive force (15). This is hypothesized to represent a response to efflux inhibition, as the cell attempts to increase expression of the efflux pump to compensate for the low level of efflux activity. Therefore, we hypothesized that measuring the promoter activity of *ramA* could act as a reporter for efflux inhibition.

The aim of this study was to identify efflux inhibitors. To do this, we developed, validated, and implemented a high-throughput screen (HTS) and used it to identify efflux inhibitors from the Prestwick Chemical Library of 1,200 molecules comprising mostly approved drugs (by the U.S. Food and Drug Administration [FDA], the European Medicines Evaluation Agency [EMEA], and other agencies) and a larger library of 47,168 compounds from F. Hoffmann-La Roche (Roche), selected for their high chemical and pharmacological diversity. Hit compounds from the screen were analyzed for efflux-inhibitory activity and the ability to potentiate the activity of antibacterials for Gram-negative bacteria.

## RESULTS

### HTS assay optimization and validation.

To determine the concentration of chlorpromazine that gave maximum induction and use as the positive control, expression of green fluorescent protein (GFP) from the *ramA* promoter was measured in cultures of Salmonella enterica serovar Typhimurium SL1344 pMW82-*ramAp* in the absence of or presence of 25, 50, 100, or 200 μg/ml chlorpromazine. To determine the optimum optical density of the culture used as the inoculum for the assays, the experiment described above was done with cultures grown to an optical density at 600 nm (OD_600_) of 0.1, 0.2, 0.45, or 0.9 as the inoculum. To validate the reproducibility of the assay, three plate readers were used (one Fluostar Optima reader and two Fluostar Omega readers). Induction of GFP expression was observed with 25, 50, and 100 μg/ml chlorpromazine (see Fig. S1a in the supplemental material). Maximum induction was achieved with 100 μg/ml chlorpromazine; however, bacterial growth was reduced at this concentration (Fig. S1b); the concentration of chlorpromazine that gave maximum induction without impacting growth was 50 μg/ml. Growth was inhibited with 200 μg/ml chlorpromazine. Changing the OD_600_ of the inoculum caused no statistically significant difference in the maximum fold change in GFP fluorescence with 50 μg/ml chlorpromazine (*n* = 10 biological replicates, *P* value = 0.69 [analysis of variance {ANOVA}]).

10.1128/mBio.01340-20.1 FIG S1 Fold level of GFP fluorescence (A) and representative growth curves (B) in the presence of various concentrations of chlorpromazine. (A) Each dot represents the average fluorescence of two biological replicates. The fold-change is relative to the no-chlorpromazine condition for the same biological replicate. (B) Line colors are as indicated in axis labels in panel A. Download FIG S1, PDF file, 0.1 MB. Copyright © 2020 Marshall et al. 2020 Marshall et al. This content is distributed under the terms of the Creative Commons Attribution 4.0 International license.

### Induction of GFP expression in the high-throughput primary screen with the *ramAp*:*gfp* reporter.

**FIG 1:**
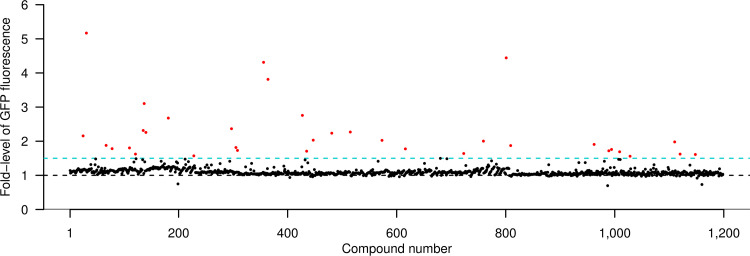
*ramAp* activity in the presence of Prestwick chemical library compounds as measured by GFP fluorescence. Each dot represents the average fluorescence of two biological replicates. The blue dashed line indicates the 1.5-fold cutoff value; the black dashed line indicates the value for the compound-free control. Hit compounds are indicated in red.

Expression of GFP from the *ramA* promoter was measured in cultures of *S.* Typhimurium SL1344 pMW82-*ramAp* in the presence of each compound in the Prestwick Chemical Library of FDA-approved drugs and the compound library from Roche. Cultures in each assay plate contained chlorpromazine and dimethyl sulfoxide (DMSO) as positive and negative controls, respectively. On the basis of data obtained previously for compounds that induced fluorescence with this reporter (15), a result showing fluorescence intensity increased to ≥1.5-fold was used to identify inducers of *ramA* expression. Of the 1,200 compounds in the Prestwick Chemical Library, 51 had increased fluorescence by >1.5-fold (Fig. 1; see also Table S1). Daunorubicin hydrochloride and merbromin were excluded from the study as they were autofluorescent. As the aim of this screen was to identify drugs that could be combined with antibiotics for use in patients, 31 of the remaining 49 compounds that induced GFP expression were eliminated from further study as they have been reported to show poor bioavailability, have a short half-life, have severe side effects, or have been characterized as affecting the bioavailability of other drugs. Cefdinir, cefixime, chlortetracycline, dequalinium dichloride, meclocycline, methacycline, minocycline, moxalactam, and oxytetracycline were also excluded as these either were known antimicrobials and/or induced filamentation due to inhibition of cell wall biosynthesis, resulting in anomalous growth readings. However, due to high induction of GFP expression (5.7-fold and 2.2-fold, respectively), chloramphenicol and tetracycline were not excluded; these are archetypical antibiotics of the amphenicol and tetracycline classes. Rifampin was also included in the study as it is not considered an effective efflux substrate (16). Nine compounds were studied further: auranofin, chloramphenicol, clofazimine, dicyclomine, dipyridamole, mefloquine, primaquine diphosphate, rifampin, and tetracycline.

10.1128/mBio.01340-20.6 TABLE S1 The 51 compounds from the Prestwick Chemical Library that caused ≥1.5-fold GFP fluorescence relative to the strain grown in MOPS minimal medium alone. Fluorescence fold-change is given as mean ± standard deviation, *n* = 2. *P* values were obtained from a Student’s *t* test for each compound with the compound-free control from the same plates. Download Table S1, DOCX file, 0.02 MB. Copyright © 2020 Marshall et al. 2020 Marshall et al. This content is distributed under the terms of the Creative Commons Attribution 4.0 International license.

On the basis of our experience with the FDA library, to reduce the number of false-positive hits in the primary screen of the Roche library of 47,168 compounds, a ≥2-fold increase in GFP fluorescence intensity was used. There were 109 compounds that caused an increase in GFP fluorescence intensity of ≥2-fold. Two compounds were subsequently excluded from further study due to their intrinsic fluorescence.

### Induction of GFP expression in a time-resolved secondary screen.

To validate the changes in fluorescence intensity from the primary screen, the assay was repeated as a time course assay. To normalize the data, specific fluorescence (units of fluorescence per unit of optical density) was calculated. The resulting GFP has a half-life of approximately 85 min (17); in the absence of continuous induction, the fluorescence from the GFP decreases. Therefore, the kinetics of induction can be measured with this reporter. From the Prestwick library, only the nine chosen compounds that caused an increase in fluorescence intensity to 1.5-fold were used in time course assays. The maximum fold induction by each compound, rather than the induction at any specified time point (Table 1), was used for comparative purposes. Three of the compounds (dipyridamole, primaquine diphosphate, and tetracycline) caused a decrease in specific fluorescence relative to the strain not exposed to any test compound (fold induction of <1.0) and were therefore not analyzed further. Although auranofin caused no change in specific fluorescence (fold induction of 1.05 ± a standard deviation of 0.05), it was included in further study as there are data to suggest that it potentiates antibiotic activity (18, 19). The remaining five compounds (chloramphenicol, clofazimine, dicyclomine, mefloquine, and rifampin) all caused an increase (1.45-fold to 2.82-fold) in specific fluorescence relative to the control.

**TABLE 1:** Fold induction of *ramAp*:*gfp* expression by Prestwick library compounds in a time course assay^*a*^

Compound	Fold induction in primary screen	Maximum fold induction of specific fluorescence in time course assay	Time at which maximum specific fluorescence was observed (min)
Auranofin	1.58	1.05 ± 0.05	102
Chloramphenicol	5.74	2.80 ± 0.11	596
Clofazimine	4.19	2.82 ± 0.47	104
Dicyclomine	1.55	1.70 ± 0.25	80
Dipyridamole	2.93	0.94 ± 0.05	51
Mefloquine	1.74	1.45 ± 0.20	75
Primaquine diphosphate	1.51	0.84 ± 0.05	83
Rifampicin	2.13	1.52 ± 0.12	590
Tetracycline	2.23	0.71 ± 0.08	494

a Values are reported as averages ± standard deviations; *n* = 6 biological replicates.

Time course assays were completed for 85 Roche compounds. Compared to the compound-free control, 44 compounds caused an increase in the specific fluorescence of GFP to ≥2-fold (Fig. S2).

10.1128/mBio.01340-20.2 FIG S2 Maximum fold induction of *ramAp*:*gfp* fluorescence by Roche compounds in the confirmatory assay. Each dot represents the average fluorescence of two biological replicates. The blue dashed line indicates the 2-fold cut-off value; the black dashed line indicates the value of the compound-free control. Download FIG S2, TIF file, 0.1 MB. Copyright © 2020 Marshall et al. 2020 Marshall et al. This content is distributed under the terms of the Creative Commons Attribution 4.0 International license.

### Off-target, non-*ramA* promoter-specific activity.

We hypothesized that some compounds might have a global effect on gene expression, or might be able to stabilize the unstable GFP and so increase GFP fluorescence when used in this assay. Therefore, the 6 remaining Prestwick library compounds and 85 Roche compounds used in time course assays were screened for nonspecific effects unrelated to *ramA* promoter-specific activity at final concentrations of 10 μM and 20 μM, respectively. This was done using time course assays with two alternative reporter constructs, *bamAp*:*gfp* and *gabDp*:*gfp*. Neither of these promoters responds to deletion or inactivation of *acrAB* or *tolC* genes or to inhibition by efflux inhibitors (20). Specific fluorescence of GFP expressed under the control of the *bamA* promoter was found to have increased (1.5-fold) only in the presence of chloramphenicol. Under the control of the *gabD* promoter, specific fluorescence of GFP was increased by chloramphenicol (1.4-fold) and rifampin (1.5-fold). None of the Roche compounds caused GFP fluorescence increases of ≥2-fold from either promoter (Fig. S3).

10.1128/mBio.01340-20.3 FIG S3 Relative GFP fluorescence in the presence of Roche compounds using *ramAp*:*gfp* (red) or *bamAp*:*gfp* (blue) constructs. Each dot represents the average fold-level of maximum fluorescence of three biological replicates. The green dashed line indicates the 2-fold cut-off value; the black dashed line indicates the value of the compound-free control. Download FIG S3, TIF file, 0.1 MB. Copyright © 2020 Marshall et al. 2020 Marshall et al. This content is distributed under the terms of the Creative Commons Attribution 4.0 International license.

### Concentration-dependent induction of pMW82-*ramAp*.

**FIG 2:**
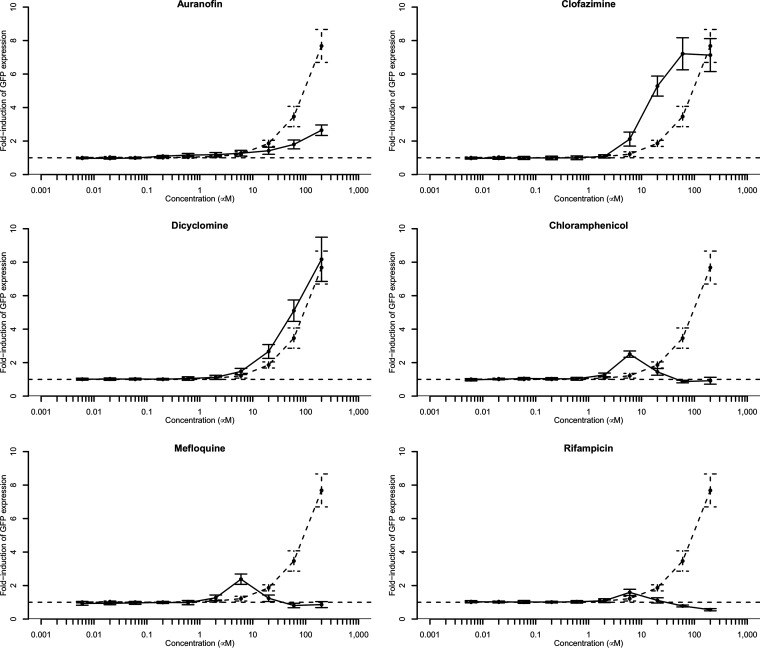
Dose response curves of GFP fluorescence from *ramA*::*gfp* in the presence of increasing concentrations of test compounds. Dashed lines, chlorpromazine; solid lines, test compound. Error bars represent standard deviations. Values were normalized to the compound-free control value.

To determine the concentration that causes maximum induction of expression from the *ramA* promoter and the time at which this occurs, time course experiments were done with *S.* Typhimurium pMW82-*ramAp* with 10 concentrations (6 nM to 200 μM) of test compounds from the Prestwick library (Fig. 2). The positive control, chlorpromazine, induced GFP production at concentrations of ≥20 μM. As the concentration of chlorpromazine increased, so did the level of fluorescence (dashed lines in Fig. 2). Dicyclomine and clofazimine showed similar dose responses to chlorpromazine, although induction of GFP expression by clofazimine started at a lower concentration (6 μM) and reached a response saturation point of 60 μM, above which increasing concentrations did not increase fluorescence. An increase in GFP expression was observed with increasing concentrations of auranofin; however, compared to chlorpromazine, the levels of induction were low within the tested concentration range. Chloramphenicol, mefloquine, and rifampin gave concentration-dependent induction between 0.6 μM and 6 μM, above which the fluorescence intensity decreased to background levels at 60 μM (at higher concentrations, these compounds had antibacterial activity). Therefore, these three compounds were excluded from further study. Auranofin, clofazimine, and dicyclomine were investigated for efflux-inhibitory properties.

A total of 66 of the 85 Roche compounds were investigated; 21 showed no dose response, and 27 reached a response saturation point above which increasing the concentrations did not increase fluorescence. For nine of the Roche compounds, there was a maximum inducing concentration above which fluorescence intensity decreased. Eighteen Roche compounds showed a dose response similar to that of chlorpromazine (data not shown). All 45 compounds that showed a dose response were studied further; 22 of the 45 compounds were later excluded due to cytotoxicity, mitochondrial toxicity, or low chemical tractability. As it was shown previously that the effects of pan-assay interference structures (PAINS) are dependent upon the wider structural context in which they occur and therefore that PAINS-containing compounds should not be excluded *a priori* (21), hit compounds were not excluded based upon PAINS analysis alone. Therefore, 23 compounds were investigated further for efflux-inhibitory effects.

### Effect of hit compounds upon ethidium bromide efflux and H33342 accumulation.

**FIG 3:**
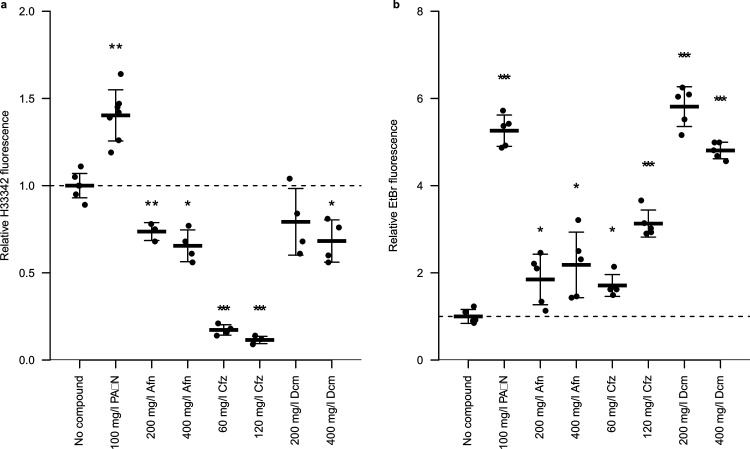
Fluorescent dye accumulation (a) and efflux (b) by *S.* Typhimurium SL1344 L in the presence of Prestwick chemical library compounds. Afn, auranofin; Cfz, clofazimine; Dcm, dicyclomine hydrochloride. Each dot represents a biological replicate. Average bars represent means. Error bars represent standard deviations. Values were normalized to the mean value of the compound-free control. Dashed lines indicate no change from the compound-free control. *, *P* < 0.05; **, *P* < 0.001; ***, *P* < 0.0001 (Student’s *t* test performed with the compound-free control).

To determine if the compounds identified as inducing *ramA* also inhibited efflux, dye accumulation assays and efflux assays were carried out for auranofin, clofazimine, dicyclomine, and 23 selected Roche compounds. The Prestwick chemical library compounds were used at concentrations equal to and double the maximum *ramAp*-inducing concentrations, as determined in dose response assays. Hoechst H33342 (bisbenzimide) and ethidium bromide are commonly used fluorescent probes, the fluorescence intensities of which increased markedly when bound to DNA. Therefore, in Hoechst H33342 accumulation assays, in which the dye is added to the extracellular environment during the assay and diffuses into the bacteria, increased fluorescence intensity correlates with increased accumulation of the dye within the cell, which negatively correlates with efflux activity. For both dyes, bacteria treated with the positive-control efflux inhibitor PAβN (phenylalanine-arginine β-naphthylamide) showed increased fluorescence intensity relative to untreated cells. The H33342 fluorescence intensity of *S.* Typhimurium SL1344 treated with auranofin, clofazimine, or dicyclomine was decreased relative to the levels seen with the untreated cells (Fig. 3a). This was particularly clear for treatment with clofazimine. In ethidium bromide efflux assays, bacteria are deenergized by treatment with carbonyl cyanide *m*-chlorophenylhydrazone (CCCP) and preloaded with ethidium bromide; glucose is added during the assay to reenergize the bacteria and thus initiate efflux, leading to a loss of ethidium bromide fluorescence. Compared with untreated cells, there was an increase in fluorescence in the presence of auranofin, clofazimine, or dicyclomine (Fig. 3b).

**FIG 4:**
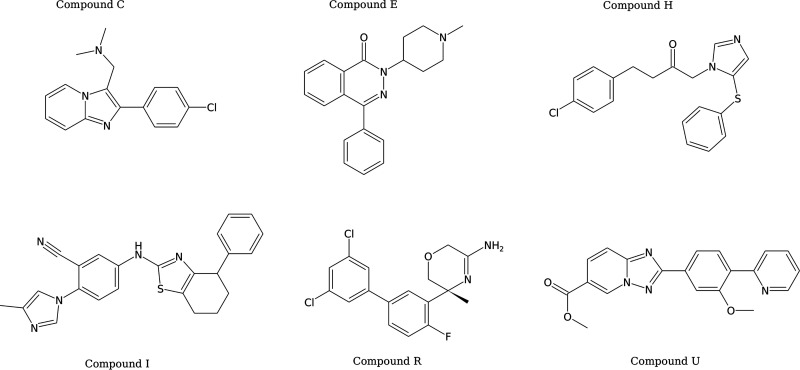
Structures of the Roche compounds used for hit expansion. Letters refer to the compound identifier. Hit expansion compounds were assigned a code that refers to the initial seed structure as indicated by the following example: for the AB1 compound, B1 represents the unique compound from the hit expansion of compound A from the initial screen (see hit expansion map in Fig. 8).

**FIG 5:**
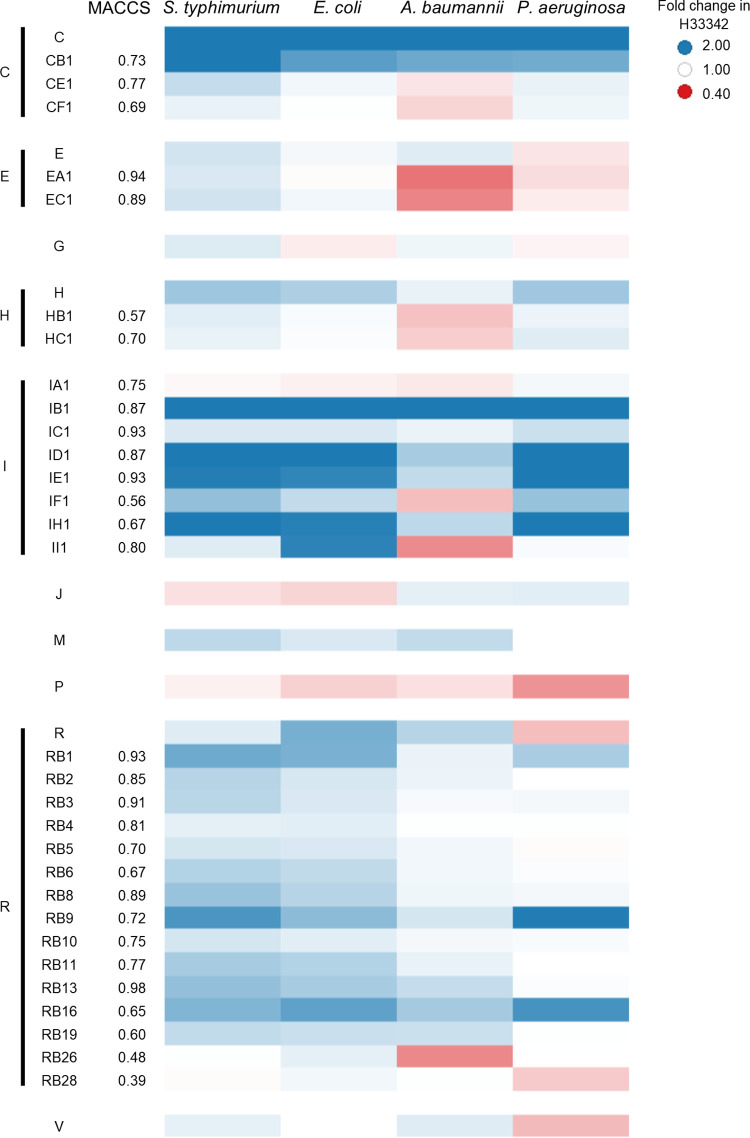
Heat map of the number of species with which H33342 accumulation was increased by Roche compounds. MACCS values represent the similarity scores calculated for individual compounds compared with the original hit compound of the same series. Increased H33342 accumulation is indicated by graduated blue coloring, whereas decreased H33342 accumulation is indicated by graduated red coloring.

Due to the relatively large amount of compound needed for the H33342 accumulation assays and ethidium bromide assays, only H33342 accumulation assays were performed with the strains of *S.* Typhimurium, Escherichia coli, Pseudomonas aeruginosa, and Acinetobacter baumannii. Of the 23 compounds (A to W) selected from the primary screen, nine (C, E, G, H, J, M, P, R, and V) increased H33342 fluorescence in at least one of the species tested (Fig. S4). To explore structure-activity relationships of 6 hits (C, E, H, I, R, and U; Fig. 4), an additional 32 compounds (CA1 to CF1, EA1 to EF1, HA1 to HC1, IA1 to II1, RA1, RB1, and UA1 to UF1) with various degrees of similarity to the initial hit compounds were selected as limited hit expansion. In H33342 accumulation assays (Fig. S5a), five of these hit expansion compounds (IB1, IC1, ID1, IE1 and IH1) increased H33342 fluorescence with all four species; five compounds (CB1, CE1, CF1, EC1, and RB1) increased fluorescence with three of the four species; two compounds (HB1 and II1) increased fluorescence with two of the four species; and four compounds (EA1, HC1, IA1, and IF1) increased fluorescence with one of the four species. The remaining 16 compounds did not increase fluorescence with any of the tested strains. Following the determination of these results and those obtained with checkerboard assays for potentiation of antibacterial drugs (see below), a further 30 compounds (RB2 to RB31) in the same hit expansion series as compound RB1 were used in H33342 accumulation assays (Fig. S5b). Compounds RB9 and RB16 increased fluorescence with all four species tested; compounds RB2, RB5, and RB6 increased fluorescence with three of the four species; compounds RB4, RB8, RB10, RB11, RB13, and RB19 increased fluorescence with two of the four species; and compounds RB3, RB26, and RB28 increased fluorescence with one of the four species. The remaining 16 compounds from the RB series of compounds did not increase fluorescence with any of the strains tested. In total, 85 compounds from Roche were used in accumulation assays, of which 8 increased H33342 fluorescence in all four species tested, 11 increased fluorescence with three of the four species, 10 increased fluorescence with two species, and 11 increased fluorescence with only one species (Fig. S5). As determined by Molecular ACCess System (MACCS) structural analysis (22), the number of species with which H33342 accumulation was increased by the compounds was unrelated to the structural similarity of the compounds (Fig. 5). However, this is unsurprising as the number of tested hit expansion compounds was limited and no clear structural activity relationship for many efflux pump substrates or inhibitors has been shown to date.

10.1128/mBio.01340-20.4 FIG S4 H33342 accumulation in the presence of Roche original hit compounds A to W. ▲, *E. coli* ATCC 25922; △, *S*. Typhimurium SL1344; ●, *P. aeruginosa* ATCC 27853; ○, *A. baumannii* AYE. Dashed line indicates no change from the compound-free control. Red symbols indicate results were statistically significantly different from the compound-free control (Student’s *t* test, *P* < 0.05). Download FIG S4, TIF file, 0.1 MB. Copyright © 2020 Marshall et al. 2020 Marshall et al. This content is distributed under the terms of the Creative Commons Attribution 4.0 International license.

10.1128/mBio.01340-20.5 FIG S5 H33342 accumulation in the presence of hit expansion compounds from Roche. ▲, *E. coli* ATCC 25922; △, *S*. Typhimurium SL1344; ●, *P. aeruginosa* ATCC 27853; ○, *A. baumannii* AYE. Dashed line indicates no change from the compound-free control. Red symbols indicate results were statistically significantly different to the compound-free control (Student’s *t* test, *P* < 0.05). Download FIG S5, TIF file, 0.3 MB. Copyright © 2020 Marshall et al. 2020 Marshall et al. This content is distributed under the terms of the Creative Commons Attribution 4.0 International license.

These data indicate that three compounds from the Prestwick chemical library and 40 compounds from the pharmaceutical company have efflux-inhibitory activity.

### Potentiation of antibiotic activity by efflux inhibitor compounds.

Checkerboard assays are a routine method by which the effects of drug combinations are assessed by creating two-dimensional concentration gradients. To determine whether the efflux-inhibitory activity of any of the compounds translated to potentiation of antibiotic activity, checkerboard assays were used to determine the extent to which the compounds potentiated the antibacterial activity of three antibiotics and a dye that are known to be substrates of efflux pumps, including AcrAB-TolC of E. coli and *S.* Typhimurium and its homologues MexAB-OprM in P. aeruginosa and AdeABC in A. baumannii. Chloramphenicol, nalidixic acid, and tetracycline were chosen as prototypical representatives of different classes of antibiotics with distinct modes of action, and the dye ethidium bromide was chosen as a well-documented nonantibiotic efflux substrate (23, 24). The known AcrB inhibitor PAβN was used as a positive control for potentiation of antibacterial activity (19, 25). Strains overexpressing MDR efflux pumps were used to maximize the sensitivity of these assays, as the amount of efflux activity lost upon inhibition should be greater if there are more copies of the pump proteins and so there should be more efflux activity when they are left uninhibited. Therefore, efflux inhibition is easier to observe.

The fractional inhibitory concentration (FIC) index of each combination indicated that auranofin potentiated the activity of tetracycline against *S.* Typhimurium and A. baumannii, of ethidium bromide against *S.* Typhimurium, and of chloramphenicol against A. baumannii (Table 2). Neither clofazimine nor dicyclomine potentiated activity of any of the tested antibiotics with any of the tested species (Table S2). Against P. aeruginosa, the combination of auranofin and chloramphenicol was antagonistic. None of the compounds with efflux-inhibitory activity from the Prestwick chemical library potentiated antibacterial activity against E. coli.

**TABLE 2:** MICs of antibiotics and ethidium bromide for *S.* Typhimurium and A. baumannii strains at the indicated concentrations of auranofin^*a*^

Strain and Afn concn (μΜ)	MIC (μg/ml)			
	Chl	Nal	Tet	EtBr
*S.* Typhimurium SL1344 *ramR*::*aph*				
0	16	8	4	512
6	16	8	**1**	256
13	16	4	**1**	**128**
25	16	4	**0.5**	**64**
50	0.06		0.015	8

A. baumannii AB211				
0	128	512	>2,048	128
6	**16**	32	**4**	**32**
13	1		2	2

a Chl, chloramphenicol; Nal, nalidixic acid; Tet, tetracycline; EtBr, ethidium bromide; Afn, auranofin. Bold font indicates synergy, as determined by an FIC index value of <0.5.

10.1128/mBio.01340-20.7 TABLE S2 MICs (μg/ml) of antibiotics and ethidium bromide for E. coli, A. baumannii, and P. aeruginosa strains at the corresponding concentration of putative efflux inhibitor (μM). Chl, chloramphenicol; Nal, nalidixic acid; Tet, tetracycline; EtBr, ethidium bromide; Afn, auranofin; Cfz, clofazimine; Dcm, dicyclomine hydrochloride. Bold font indicates synergy, as determined by an FIC index of <0.5. Download Table S2, DOCX file, 0.02 MB. Copyright © 2020 Marshall et al. 2020 Marshall et al. This content is distributed under the terms of the Creative Commons Attribution 4.0 International license.

**FIG 6:**
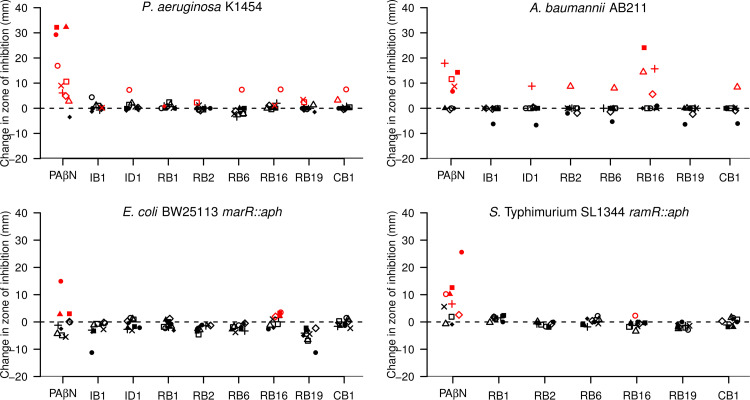
Change in size of the zone of growth inhibition (mm) of the indicated species around antibiotic-containing discs in the presence of putative efflux inhibitors. □, cefotaxime; ■, chloramphenicol; ▲, nalidixic acid; △, meropenem; ◆, streptomycin; ◇, polymyxin B; ○, ciprofloxacin; ●, erythromycin; ＋, piperacillin-tazobactam; X, tetracycline. Red symbols indicate that results were statistically significantly different from those obtained with the compound-free control (*P* < 0.05 [Student’s *t* test]).

Of the primary hit and expansion compounds from Roche, nine were tested in checkerboard assays. One compound potentiated the activity (defined by an FIC index value of <0.5) of chloramphenicol and nalidixic acid against A. baumannii AB211 and *S.* Typhimurium SL1344 *ramR*::*aph*, respectively (Table S3). On the basis of this result, 30 hit expansion compounds of RB1 were provided. As data from the H33342 accumulation assays suggested that additional compounds possessed efflux-inhibitory activity, eight compounds from the initial hit expansion and the RB series of hit expansion compounds were investigated for potentiation of antibiotic activity. Given that checkerboard assays rely upon doubling dilutions of both the antibiotic and compound of interest, small yet significant differences may be missed, particularly between high-concentration dilutions. Therefore, disc diffusion assays performed with the compound of interest incorporated into the agar and the antibiotics applied in discs were used to allow a continuous gradient of antibiotic concentration to be tested against a single concentration (60 μM) of putative efflux inhibitor. In disc diffusion assays, all of the compounds tested increased the size of the zone of inhibition for at least one antibiotic with P. aeruginosa K1454 that overproduced MexAB-OprM (Fig. 6). At 60 μM, compound RB1 inhibited growth of A. baumannii AB211 that overproduced AdeABC; a broth microdilution MIC assay confirmed that the MIC of this compound is 60 μM against this strain. The size of the zone of inhibition against AB211 was increased for one antibiotic by each of compounds CB1, ID1, RB2, and RB6 and for four antibiotics by compound RB16. Tested with either E. coli or *S.* Typhimurium, only compound RB16 caused any increase in the size of the zones of inhibition.

10.1128/mBio.01340-20.8 TABLE S3 MICs (μg/ml) of antibiotics and ethidium bromide for E. coli, A. baumannii, and P. aeruginosa strains at the corresponding concentration of putative efflux inhibitor RB1 (μM). Chl, chloramphenicol; Nal, nalidixic acid; Tet, tetracycline; EtBr, ethidium bromide; Afn, auranofin; Cfz, clofazimine; Dcm, dicyclomine hydrochloride. Bold font indicates synergy, as determined by an FIC index of <0.5. Download Table S3, DOCX file, 0.01 MB. Copyright © 2020 Marshall et al. 2020 Marshall et al. This content is distributed under the terms of the Creative Commons Attribution 4.0 International license.

To determine the concentrations of antibiotics and test compounds that have the greatest effect on antibiotic activity, the compounds which caused the most potentiation in disc diffusion assays were tested in checkerboard assays in combination with a variety of antibiotics from the classes for which they potentiated activity in disc diffusion assays. Compound CB1 was tested in combination with fluoroquinolones and carbapenems, compounds IB1 and RB6 with fluoroquinolones only, compound RB16 with fluoroquinolones and tetracyclines, and compound RB19 with carbapenems and tetracyclines. Individual antibiotics within each class were chosen to maximize the structural diversity of the combination antibiotics. In checkerboard assays, compounds IB1, RB6, and RB19 did not potentiate the activity of any of the antibiotics with which they were tested in checkerboard assays. However, while compound RB16 did not potentiate activity of minocycline or the fluoroquinolones, it potentiated the activity of doxycycline, tigecycline, and demeclocycline (Table 3). Compound CB1 potentiated the activity of ciprofloxacin.

**TABLE 3:** MICs of antibiotics for P. aeruginosa strain K1454 at the indicated concentrations of putative efflux inhibitor^*a*^

Compoud and concn (μM)	MIC (μg/ml)										
CB1	Cip	Nor	Mxf	Oxo	Tva	Fin	Dlx	Mem	Ipm	Dor	Etp
0	0.25	1	2	32	2	8	2	2	0.5	0.5	32
4	0.12	0.5	1	16	1	4	2	1	0.25	0.25	32
8	0.12	0.5	1	16	1	4	2	1	0.25	0.25	32
16	0.12	0.5	1	16	1	4	2	1	0.25	0.25	16
32	0.12	0.5	2	16	1	4	2	1	0.25	0.25	32
64	0.12	0.5	1	16	1	4	2	1	0.25	0.25	32
128	**0.06**	0.5	1	16	1	4	2	2	0.25	0.25	32
256	0.12	0.5	1	32	1	8	2	2	0.5	0.25	64

RB16	Cip	Nor	Mxf	Oxo	Tva	Fin	Dlx	Dox	Min	Tgc	Dmc
0	0.5	1	4	64	2	16	2	128	64	64	64
4	0.25	1	2	32	2	8	1	64	32	32	32
8	0.25	1	2	32	2	8	2	64	32	32	32
16	0.25	1	2	32	2	8	2	64	32	32	32
32	0.25	1	2	32	2	16	2	64	32	32	**16**
64	0.25	1	2	32	2	16	2	**32**	32	32	**16**
128	0.25	1	2	32	2	8	2	**32**	32	**16**	**16**
256	0.25	1	2	32	2	16	2	**32**	32	**16**	**16**

RB6	Cip	Nor	Mxf	Oxo	Tva	Fin	Dlx				
0	0.5	2	4	64	2	16	4				
4	0.25	1	2	32	1	8	**1**				
8	0.25	2	2	32	1	8	**1**				
16	0.25	1	2	32	1	8	2				
32	0.25	1	2	64	1	8	**1**				
64	0.25	2	2	64	2	16	2				
128	0.25	2	4	64	2	16	2				
256	0.5	2	4	64	2	16	4				

IB1	Cip	Nor	Mxf	Oxo	Tva	Fin	Dlx				
0	0.5	2	4	64	2	16	4				
4	0.25	1	2	32	1	8	**1**				
8	0.25	1	2	32	1	8	**1**				
16	0.25	1	2	32	1	8	**1**				
32	0.25	1	2	32	1	16	2				
64	0.25	1	2	32	1	16	2				
128	0.25	2	4	64	2	16	2				
256	0.5	2	4	64	2	32	4				

a Cip, ciprofloxacin; Nor, nofloxacin; Mxf, moxifloxacin; Oxo, oxolinic acid; Tva, trovafloxacin; Fin, finafloxacin; Dlx, delafloxacin; Mem, meropenem; Ipm, imipenem; Dor, doripenem; Etp, ertapenem; Dox, doxycycline; Min, minocycline; Tgc, tigecycline; Dmc, demeclocyline. Bold font indicates synergistic interactions as determined by an FIC index of <0.5.

**FIG 7:**
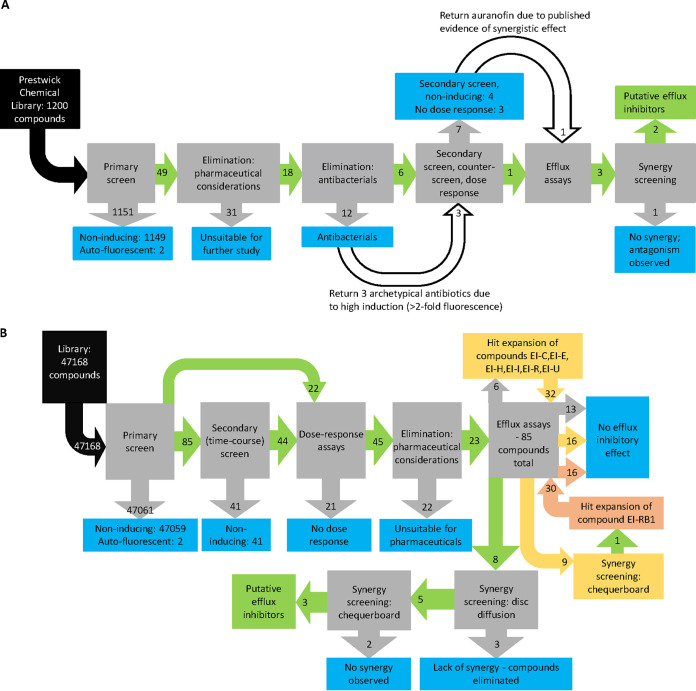
(A) Flowchart of processes and elimination of compounds from the Prestwick chemical library. (B) Flowchart of processes and elimination of Roche compounds.

Following exclusion of compounds based on toxicity and drug interaction properties, 88 compounds were screened for efflux-inhibitory properties by Hoechst 33342 uptake and ethidium bromide efflux assays. From this process (summarized in Fig. 7), 43 compounds were identified as putative efflux inhibitors, 11 of which, including auranofin and dicyclomine from the Prestwick chemical library, potentiated antibiotic activity.

To determine if the lead compounds identified in this study were able to reduce the MIC values of selected antibiotics, we compared MIC values of the same antibiotics in mutants lacking a functional RND pump. Unfortunately, MIC data for the antibiotics tested in this study and a P. aeruginosa *mexB* deletion mutant or A. baumannii *adeB* deletion mutant were not available. However, in a *S.* Typhimurium and E. coli *acrB* deletion mutant and an AcrB nonfunctional mutant, the MIC values of the antibiotics tested were found to have decreased by a magnitude of 2-fold to 4-fold more than the reductions seen in the presence of the efflux inhibitor compounds (data not shown). These data suggest incomplete inhibition of transport via RND efflux pumps.

We also compared the values obtained with the efflux inhibitors to the recommended breakpoint concentrations. According to EUCAST Clinical Breakpoint Tables v. 10.0, valid from 1 January 2020 (https://www.eucast.org/fileadmin/src/media/PDFs/EUCAST_files/Breakpoint_tables/v_10.0_Breakpoint_Tables.pdf), there are no defined clinical breakpoint concentrations for chloramphenicol, tetracycline, or nalidixic acid for Acinetobacter spp. and *Pseudomonas* spp. For the *Enterobacterales*, the clinical breakpoint concentration for chloramphenicol is 8 μg/ml; there are none for tetracycline or nalidixic acid. The inhibitors identified in this study reduced the MIC value of chloramphenicol below the defined clinical breakpoint for *Salmonella* and E. coli.

### Mechanism of action of putative efflux inhibitors.

**FIG 8:**
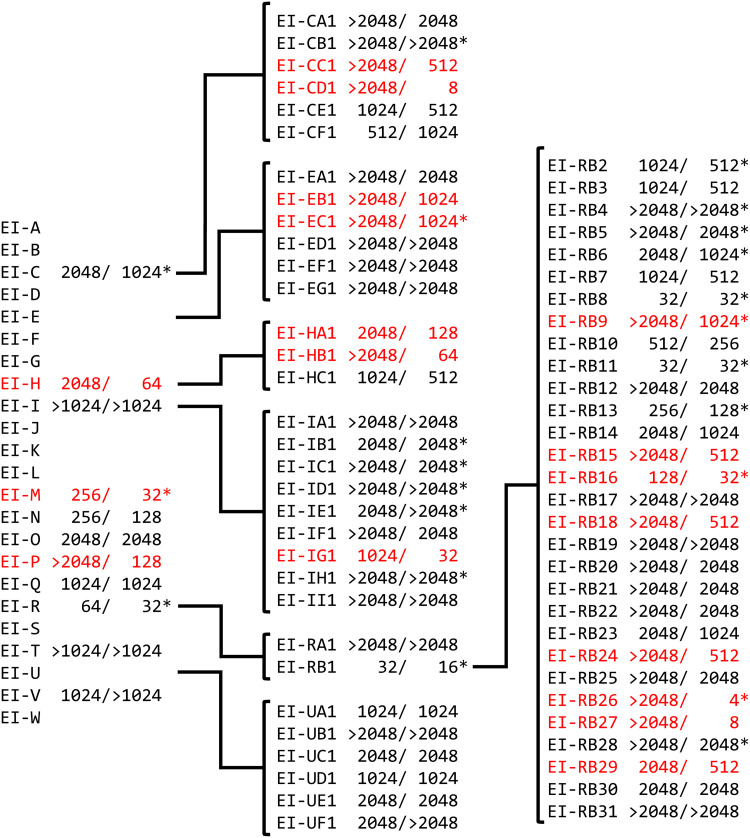
Hit expansion map of pharmaceutical company compounds and the MIC of the compounds with MG1655 and MG1655 AcrB D408A. MICs are indicated in micromolar (μM), first for MG1655 and then for MG1655 AcrB D408A. Red coloring indicates compounds for which the MIC was lower for the strain lacking functional AcrB than for the wild-type strain. *, Hoechst H33342 accumulation was increased in the presence of the indicated compound.

As other efflux inhibitors, including chlorpromazine and PAβN, are substrates of AcrB (26, 55), we sought to determine if the efflux inhibitors identified by this study are also substrates of the efflux pumps. If a compound is a substrate only of AcrB, then loss of AcrB function via a mutation conferring D408A causes hypersusceptibility to the compound. The MICs of 73 Roche compounds were tested with MG1655 and MG1655 AcrB D408A. For 20 compounds, the mutant was more susceptible than the wild type (Fig. 8). The MIC was higher for the mutant than for the wild type for one compound. For 23 compounds, there was no measurable MIC for the wild-type strain. These data suggest that 43 of the 73 compounds are AcrB substrates. The MICs of nine of the compounds were the same for both strains. For 16 compounds and both strains, and for 3 compounds with the mutant strain only, the MIC was greater than the maximum tested concentration. There was no correlation between the fold level of H33342 accumulation in the presence of a compound and the number of doubling dilution differences in MIC for the two strains (Pearson’s *R* = 0.0423, calculated using only compounds for which the MIC was measurable with both strains).

There was a difference of greater than 2 orders of magnitude in the MICs of compounds CE1, RB26, and RB27 for the mutant versus the wild type. This suggests that AcrB activity is particularly important for the intrinsic resistance to these compounds. Compound RB16, which caused most potentiation of antibiotic activity, had an MIC of 128 μM for the wild-type strain and an MIC of 32 μM for the mutant lacking AcrB activity, suggesting that this compound may be a “preferential substrate” over the tested antibiotics, similarly to the mode of action proposed for chlorpromazine (55).

## DISCUSSION

Several companies have or have had drug discovery programs aimed at finding inhibitors of multidrug efflux that could be used as an adjunct in antimicrobial chemotherapy, including TAXIS Pharmaceuticals (https://carb-x.org/carb-x-news/carb-x-funds-taxis-pharmaceuticals-to-accelerate-development-of-innovative-efflux-pump-inhibitors-epis-a-new-drug-class-that-would-impair-bacterias-ability-to-fight-antibiotics/), Pfizer (27), Microbiotix (28), ReaLi Tide Biological Technology (29), Daiichi, and Microcide (19, 25, 30). To date, none of the inhibitors identified have been developed, predominantly due to toxicity issues (31). Most discovery programs to identify efflux inhibitors have utilized checkerboard assays (19, 29, 30), potentiation of antibiotic activity at a single subinhibitory concentration (25), or dye uptake assays (27, 32). We have developed a screen to identify efflux inhibitors based upon the hypothesis that exposure to an efflux inhibitor causes an increase in *Salmonella ramA* expression (15).

By using a *ramAp*:*gfp* reporter construct, which responds to efflux inhibition without addition of antibiotics, and regardless of the method of inhibition, we developed a high-throughput screen that successfully identified inhibitors of multidrug efflux from two libraries of compounds. Three of the identified compounds from the Prestwick library have been previously identified as efflux inhibitors in bacteria (33–35), thus validating the screen, and a further 40 from the Roche library were characterized as inhibitors of multidrug efflux in Gram-negative bacteria. A further four of the identified compounds from the Prestwick library have been reported to potentiate antibiotic activity but without a mechanism being identified (18, 36), while four other compounds identified by our screen are known to inhibit transport in eukaryotic cells (32, 37–39).

From a total of 48,368 compounds in the two libraries, the primary screen identified 157 compounds that induced expression of *gfp* from the *ramA* promoter and were classed as putative efflux inhibitors. Further investigation revealed that 43 inhibited efflux of one or both dyes and that 11 potentiated the activity of the tested antibiotics for one or more of the strains of E. coli, P. aeruginosa, A. baumannii or *S.* Typhimurium that overexpressed AcrB or its homologue. As some of the Roche compounds were more effective in combination with specific antibiotics, we hypothesize that efflux inhibitors can be substrate and species specific; for example, doxycycline, tigecycline, and demeclocycline were potentiated by compound RB16 but minocycline was not. Furthermore, E. coli cells lacking a functional AcrB (due to a D408A substitution inhibiting the proton relay of AcrB) were more susceptible to some of the efflux inhibitors than the wild-type cells, suggesting that they are substrates of AcrB. A recent molecular dynamics study revealed that amitriptyline and chlorpromazine are also efflux substrates that appear to inhibit efflux of other compounds (55). The molecular dynamics study also indicated that amitriptyline and chlorpromazine have different efflux-inhibitory effects in combination with ethidium bromide or norfloxacin due to the differences in binding locations within AcrB of the inhibitors and substrates. We postulate that the same is true for the Roche efflux inhibitors.

For nine of the Roche compounds validated as efflux inhibitors, there was no difference in the MICs for the two E. coli strains; it was beyond the scope of this study to determine the mechanism by which these compounds inhibit efflux activity, but it could be that the compounds inhibit pumps other than AcrB. It is also possible that some of the identified efflux inhibitors for which little or no potentiation was observed with the tested drugs may potentiate the activity of antibiotics that were not tested or may inhibit pumps that do not transport antibiotics. It was beyond the scope of this study to identify all of the antibiotics and species for which each of the efflux inhibitors potentiates antibiotic activity.

Analysis of data for hits from the Prestwick library revealed that in dose response assays, the decrease in fluorescence intensity at higher concentrations of rifampin and chloramphenicol was most likely due to the compounds inhibiting mRNA and protein synthesis, respectively, at concentrations exceeding the MIC (each approximately 12 μM). Following dose response assays, mefloquine was also excluded from further investigation as it caused a decrease in GFP fluorescence at higher concentrations (>20 μM). The MIC of mefloquine against E. coli has previously been reported to be 56 μM (40); thus, the decrease in GFP fluorescence at higher concentrations of mefloquine may be due to the antibacterial activity of this drug. Mefloquine was previously identified as an efflux inhibitor with activity against both E. coli and P. aeruginosa (35), although it had earlier been reported to disrupt the membranes of E. coli (40). Dicyclomine is an anticholinergic drug that is used in the treatment of irritable bowel syndrome and prevents the methamphetamine-induced efflux of dopamine through eukaryotic transporter proteins (38). Our data suggest that dicyclomine is an efflux inhibitor. The antirheumatism drug auranofin has been reported to have broad-spectrum antibacterial activity and to synergize with other antibiotics by inhibiting thioredoxin reductase (18). Our data suggest that the synergistic effect of auranofin also may be due to inhibition of an efflux pump. Other drugs identified from the Prestwick chemical library by the primary screen, but not investigated further, include prenylamine, depridil, and fendiline, all of which are Ca-blocking drugs. Interestingly, deenergizing the cell by blocking of calcium-dependent processes is hypothesized to be a part of the mode of action of phenothiazine-mediated efflux inhibition in both eukaryotic and prokaryotic cells (41). Chlorprothixene, methiothepin, and thioridazine are antipsychotic drugs and were also hits in the primary *ramAp* reporter screen; thioridazine has previously been shown to inhibit efflux activity in Gram-negative bacteria (33).

A study by Hind et al. used the Prestwick Chemical Library to identify compounds that overcome multidrug resistance by any mechanism (36). Of the 14 compounds identified as “antibiotic resistance breakers,” four (auranofin, daunorubicin, thioridazine, and zidovudine) were also identified by the *ramAp*:*gfp* reporter screen. Auranofin, clofazimine, and dicyclomine increased ethidium bromide retention, suggesting that they are probably efflux inhibitors; however, accumulation of H33342 was unaffected, showing a substrate-dependent effect. Among the compounds that were identified by Hind et al. and were not hits in our *ramAp*:*gfp* screen, we hypothesize that they potentiate antibiotic activity by a mechanism unrelated to efflux inhibition. We also hypothesize that the compounds identified by our *ramAp*:*gfp* screen but not by Hind et al. inhibit the efflux of antibiotics that were not tested in the study by Hind et al.

Our screen of the Prestwick chemical library screen identified several efflux inhibitors, among which only auranofin potentiated antibiotic activity in checkerboard assays. Therefore, data arising from experiments performed with the hit compounds from the Prestwick chemical library screen may be of interest for chemical expansion studies to identify derivatives that have greater efflux-inhibitory activity and that can be used in computational studies to identify the mechanism by which they inhibit efflux. This will help in the intelligent design of novel efflux inhibitors.

In conclusion, the new high-throughput screen is a valuable tool to identify efflux inhibitors, as evidenced by the 43 (three Prestwick library compounds and 40 Roche compounds) new efflux inhibitors described here.

## MATERIALS AND METHODS

### Strains and plasmids used in this study.

Wild-type Salmonella enterica serovar Typhimurium SL1344 was used as a template strain for cloning (42). The *gabD* and *bamA* promoters (*gabDp* and *bamAp*) from *S.* Typhimurium SL1344 were cloned into promoter-trap vector pMW82 (which contains the promoterless gene *gfp*) using the BamHI and XbaI restriction sites (43); the *ramA* promoter (*ramAp*) reporter had been previously constructed (44). *S.* Typhimurium SL1344 was transformed with pMW82-*ramAp*, pMW82-*bamAp*, or pMW82-*gabDp* reporters and used in fluorescence assays. Escherichia coli strain MG1655 AcrB D408A was made by chromosomal mutagenesis of the MG1655 strain, as described for the equivalent mutation in SL1344 (20). The wild-type strains *S.* Typhimurium SL1344, Acinetobacter baumannii strain AYE (45), E. coli strain ATCC 25922, and Pseudomonas aeruginosa strain ATCC 27853 were used in dye accumulation and efflux assays; the efflux pump-overexpressing strains *S.* Typhimurium SL1344 *ramR*::*aph*, A. baumannii AB211, E. coli BW25113 *marR*::*aph*, and P. aeruginosa K1454 were used in checkerboard and disc diffusion assays. E. coli strain MG1655 and its AcrB D408A mutant were used for single-compound susceptibility testing (Table 4). For routine culture of bacteria, Lennox broth (Oxoid) was used. MOPS (morpholinepropanesulfonic acid) minimal medium (Teknova) was supplemented with 0.04% (wt/vol) l-histidine to support the growth of SL1344 and its derivatives.

**TABLE 4:** Strains used for checkerboard and disc diffusion assays

Strain	Phenotype	Use	Reference or source
*S.* Typhimurium SL1344	Wild type	GFP assays, dye efflux, and accumulation	42
*S.* Typhimurium SL1344 *ramR*::*aph*	Overexpresses RamA and AcrAB	Antibiotic potentiation	50
E. coli ATCC 25922	Clinical isolate used as a quality control strain	Dye efflux and accumulation	ATCC
E. coli BW25113 *marR*::*aph*	Keio collection strain JW52481-1; overexpresses MarA and AcrAB	Antibiotic potentiation	51
E. coli MG1655	K-12 derivative regarded as wild type	Susceptibility testing	52
E. coli MG1655 AcrB D408A	Chromosomal missense mutant of MG1655 in which AcrB is inactive	Susceptibility testing	This study
A. baumannii AYE	Clinical isolate that expresses β-lactamase VEB-1	Dye efflux and accumulation	45
A. baumannii AB211	Clinical isolate that overexpresses AdeABC	Antibiotic potentiation	53
P. aeruginosa ATCC 27853	Clinical isolate used as a quality control strain	Dye efflux and accumulation	ATCC
P. aeruginosa K1454	Spontaneous *nalC* mutant of PAO1; overexpresses MexAB-OprM	Antibiotic potentiation	54

### Compounds screened and investigated for efflux-inhibitory activity.

The 1,200 FDA-approved compounds from the Prestwick Chemical Library (Prestwick Chemical, Illkirch, France) were stored as primary stock solutions at 10 mM in 100% dimethyl sulfoxide (DMSO) at –20°C. A library of 47,168 compounds was provided as blind samples at 4 mM in 100% DMSO, in 384-well plates by F. Hoffmann-La Roche. Hit compounds from Roche that were further investigated were assigned a letter to indicate the hit compound from the library. Sixty-two hit expansion compounds from the same chemical classes as the putative efflux inhibitor initial hits from the GFP assay were also investigated for efflux-inhibitory activity in dye accumulation assays. Hit expansion compounds were assigned a code, e.g., AB1, in which B1 indicates the unique compound from the hit expansion of compound A from the initial screen.

### Assay development.

The HTS assay was based upon the GFP reporter assay for promoter activity of *S.* Typhimurium *ramA* described in 2013 (15). Overnight cultures of strain SL1344 pMW82-*ramAp* were diluted to 4%, 2%, 1%, and 0.5% in MOPS medium and grown to OD_600_ levels of 0.9, 0.45, 0.2, and 0.1, respectively. Equal volumes of culture and MOPS medium with chlorpromazine at final concentrations of 200, 100, 50, and 25 mg/liter with DMSO at a final concentration of 0.4% were added to clear-bottomed, black-sided 96-well assay plates. MOPS medium with 0.4% DMSO was used as the reference condition. A control lacking inoculum (sterile MOPS medium added in place of culture) was used to blank-correct the samples during data processing. Both fluorescence of GFP (excitation and emission wavelengths of 492 nm and 520 nm, respectively) and OD_600_ were measured every 3 min for 18 h on a FLUOstar Optima or FLUOstar Omega plate reader (BMG Labtech). For each overnight culture, every combination of inoculum density and chlorpromazine concentration were tested.

### Primary screen for efflux-inhibitory activity.

An automatic liquid handling system (Microlab Star; Hamilton) was used for all liquid handling. Strain SL1344 pMW82-*ramAp* was grown to an OD_600_ of 0.9 in MOPS medium. Experiments were performed at a final volume of 200 μl in clear-bottomed, black-sided 96-well assay plates at a final concentration of 10 μM in 0.75% DMSO with a 50% dilution of the bacterial culture. Roche compounds were used at a final concentration of 20 μM in 0.4% DMSO. In all assay plates, four replicates of the negative control (DMSO alone) and the positive control (chlorpromazine at a final concentration of 50 μg/ml [140.7 μM]) (15) were included to calculate Z-prime; where Z-prime values were less than 0.5, the plate assay was repeated. Fluorescence of GFP was measured on a FLUOstar Omega plate reader approximately every hour.

### Counterscreen and kinetics of induction of GFP production.

As a measure of off-target effects (non-efflux-inhibitory activity or generally increased gene expression), two further reporter assays were used. *bamAp* was used to measure responses to membrane stress, while *gabDp* was used to report on metabolic stress. The two genes *bamA* and *gabD* had been shown previously not to respond to inactivation or deletion of *acrB* (20).

Overnight cultures of the reporter strains SL1344 pMW82-*ramAp*, SL1344 pMW82-*bamAp*, and SL1344 pMW82-*gabDp* were used to inoculate 20 ml MOPS medium at a 4% inoculum concentration and were incubated at 37°C (200 rpm) until the OD_600_ reached approximately 0.9 before being diluted by addition of 16 ml MOPS medium. Compounds of interest were dissolved in DMSO at a concentration of 10 mM and were diluted to 100 μM in MOPS medium. A 90-μl volume of diluted culture and 10-μl volumes of diluted test compounds were added to wells in round-bottomed, black-sided 96-well plates; the compounds were used at a final concentration of 10 μM (Prestwick library) or 20 μM (Roche library) in 0.4% DMSO. Both fluorescence and OD_600_ were measured every 3 min for 18 h on a FLUOstar Optima or FLUOstar Omega plate reader. Fluorescence was normalized to units of fluorescence per unit of absorbance (fluorescence/OD_600_).

### Dose response assays.

A 3-fold dose response serial dilution was performed to give a final compound concentration range of 200 to 0.006 μM. The contents of each well were further diluted 1 in 10 with the addition of SL1344 pMW82-*ramAp* culture. A final volume of 100 μl and final 200, 60, 20, 6, 2, 0.6, 0.2, 0.06, 0.02, and 0.006 μM concentrations of the test compounds in 0.4% DMSO were used. Fluorescence and OD_600_ were measured over time as indicated in the description of the assays performed to analyze kinetics of induction of GFP production.

### Dye accumulation and efflux assays.

Increases in Hoechst H33342 fluorescence in *S.* Typhimurium SL1344 were used as an indicator of efflux activity, as previously described (23, 46). For the Roche compounds, E. coli strain ATCC 25922, A. baumannii strain AYE, and P. aeruginosa strain ATCC 27853 were also used. Briefly, test compounds from the Prestwick chemical library were used at final concentrations of the maximum *ramAp*-inducing concentration (determined from dose response assays) and at double this concentration, with a final DMSO concentration of 0.4% in each well. Compounds from Roche were screened at a final concentration of 60 μM, also in 0.4% DMSO. The known efflux inhibitor phenylalanine-arginine-β-naphthylamide (PAβN) was used at a final concentration of 100 μg/ml as a positive control for *S.* Typhimurium and E. coli and at 50 μg/ml for A. baumannii and P. aeruginosa (46, 47). An overnight bacterial culture maintained in Lennox broth was used to inoculate fresh Lennox broth and then incubated aerobically at 37°C to an OD_600_ of between 0.45 and 0.60, at which point the cultures were in mid-exponential phase. Cells were harvested by centrifugation and resuspended to an OD_600_ of 0.1 in phosphate-buffered saline. Resuspended cells were added to clear-bottomed black-sided 96-well plates with the test compounds to reach a 180-μl final volume. Fluorescence was measured at 37°C with excitation and emission at 355 nm and 465 nm, respectively, every minute for 90 min on a FLUOstar Optima plate reader. After 5 measurements, 20 μl Hoechst H33342 was added to reach a final concentration of 2.5 μg/ml in each well using the injector function on the plate reader.

A decrease in ethidium bromide fluorescence in cells preloaded with the dye was used as a direct indicator of efflux activity, with modifications from the method described previously by Paixao et al. (24). Test compounds were used at the maximum *ramAp*-inducing concentration and at double this concentration, with a final DMSO concentration of 0.4% in each well. PAβN was used at a final concentration of 100 μg/ml as a positive control. An overnight culture of *S.* Typhimurium SL1344 in Lennox broth was used to inoculate fresh Lennox broth and then incubated aerobically at 37°C to reach an OD_600_ of between 0.45 and 0.60, at which point the cultures were in mid-exponential phase. Cells were harvested by centrifugation and resuspended with an OD_600_ adjustment to 0.2 in potassium phosphate buffer (pH 7.0) supplemented with 100 μM carbonyl cyanide *m*-chlorophenylhydrazone (CCCP) and 50 μg/ml ethidium bromide to preload the cells. After 30 min of incubation at room temperature, cells were harvested by centrifugation and resuspended to an OD_600_ of 0.1 in potassium phosphate buffer. Preloaded cells were added to clear-bottomed black-sided 96-well plates with the test compounds to reach a 195-μl final volume. Fluorescence was measured at 37°C, with excitation and emission at 544 nm and 590 nm, respectively, every minute for 60 min on a FLUOstar Optima plate reader. After 5 measurements, 5 μl of glucose solution was added to reach a final concentration of 25 mM in each well using the injector function on the plate reader.

### Measurement of potentiation of antibiotic activity by test compounds.

All dilutions of test compounds and antibacterial agents were made in Iso-Sensitest broth (Oxoid). Test compounds and antibacterial agents that are known substrates of MDR efflux pumps were diluted in Iso-Sensitest broth to four times the required final concentration. Checkerboard 96-well plates comprised putative efflux inhibitor test compound dilutions made in each row and antibacterial drug dilutions made in each column. Final concentrations were compound and antibiotic specific, based on the individual MICs for each test strain. Plates were incubated aerobically at 37°C for 16 to 20 h. The endpoint absorbance at 650 nm was measured on a FLUOstar Optima plate reader, and the 80% inhibitory concentration (IC_80_) was used for calculation of the FIC values.

The EUCAST disc diffusion assay was modified to incorporate putative efflux inhibitors in the agar plate (48). Iso-Sensitest agar was used instead of Müller-Hinton agar, in accordance with the British Society of Antimicrobial Chemotherapy protocol (49). Putative efflux inhibitors were added to molten Iso-Sensitest agar at 45 to 55°C to reach a final concentration of 60 μM (test compound) or 100 μg/ml (PAβΝ) before 25-ml agar plates were poured. All plates were dried for 5 min at 60°C. Suspensions of the efflux pump-overexpressing strains were made by inoculating 4 to 5 colonies in 3 ml sterile 0.85% saline solution and adjusting the turbidity to 0.5 McFarland units. Two agar plates were inoculated per cell suspension by spreading the cell suspension from a saturated sterile cotton swab. Antibiotic discs were applied to the surface of the plates within 15 min of inoculation with bacteria. The antibiotics tested were nalidixic acid (30 μg), ciprofloxacin (1 μg), tetracycline (30 μg), chloramphenicol (10 μg), and cefotaxime (5 μg) on plate “A” and piperacillin-tazobactam (30 μg), erythromycin (15 μg), meropenem (10 μg), polymyxin B (300 units), and streptomycin (10 μg) on plate “B.” Each plate also contained an antibiotic-free disc. Due to limited compound availability, two of the Roche compounds (IB and ID) were tested only in duplicate against E. coli, P. aeruginosa, and A. baumannii. Otherwise, three biological replicates of each of the four strains were used and the zones of inhibition for each antibiotic were read using a zone-reading machine (ProtoCOL3 plus; Synbiosis) after 18 h of incubation at 37°C.

### Susceptibility testing with Roche compounds.

MICs of the compounds were determined by broth microdilution in clear-sided, round-bottomed 96-well plates, with a maximum final concentration of 2,048 μM. Overnight cultures of MG1655 and MG1655 AcrB D408A were diluted 1:2,000 in Müller-Hinton broth and immediately used to inoculate wells containing equal volumes of the compounds at double their final concentration in Müller-Hinton broth. After incubation at 37°C for 16 h, the optical density at 600 nm (OD_600_) was measured on a FLUOstar Optima reader. The MIC was defined as the lowest concentration required to decrease the final OD_600_ by 80% compared to the compound-free control.
