# Supplementary figures and images for: New Multidrug Efflux Inhibitors for Gram-Negative Bacteria
Source: mBio. 2020 Jul 14;11(4):e01340-20. doi: 10.1128/mBio.01340-20 (PMC7360932; doi:10.1128/mBio.01340-20)

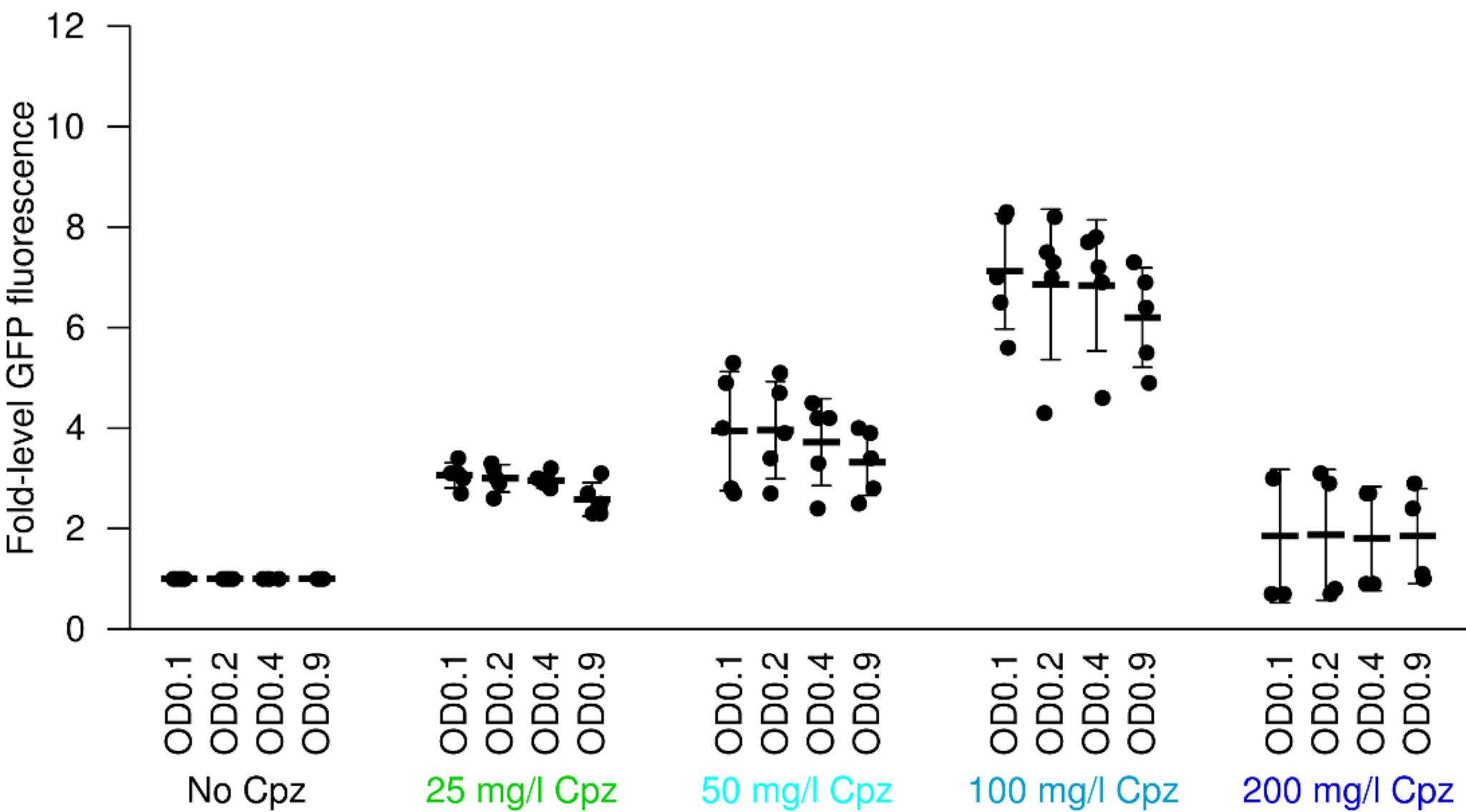

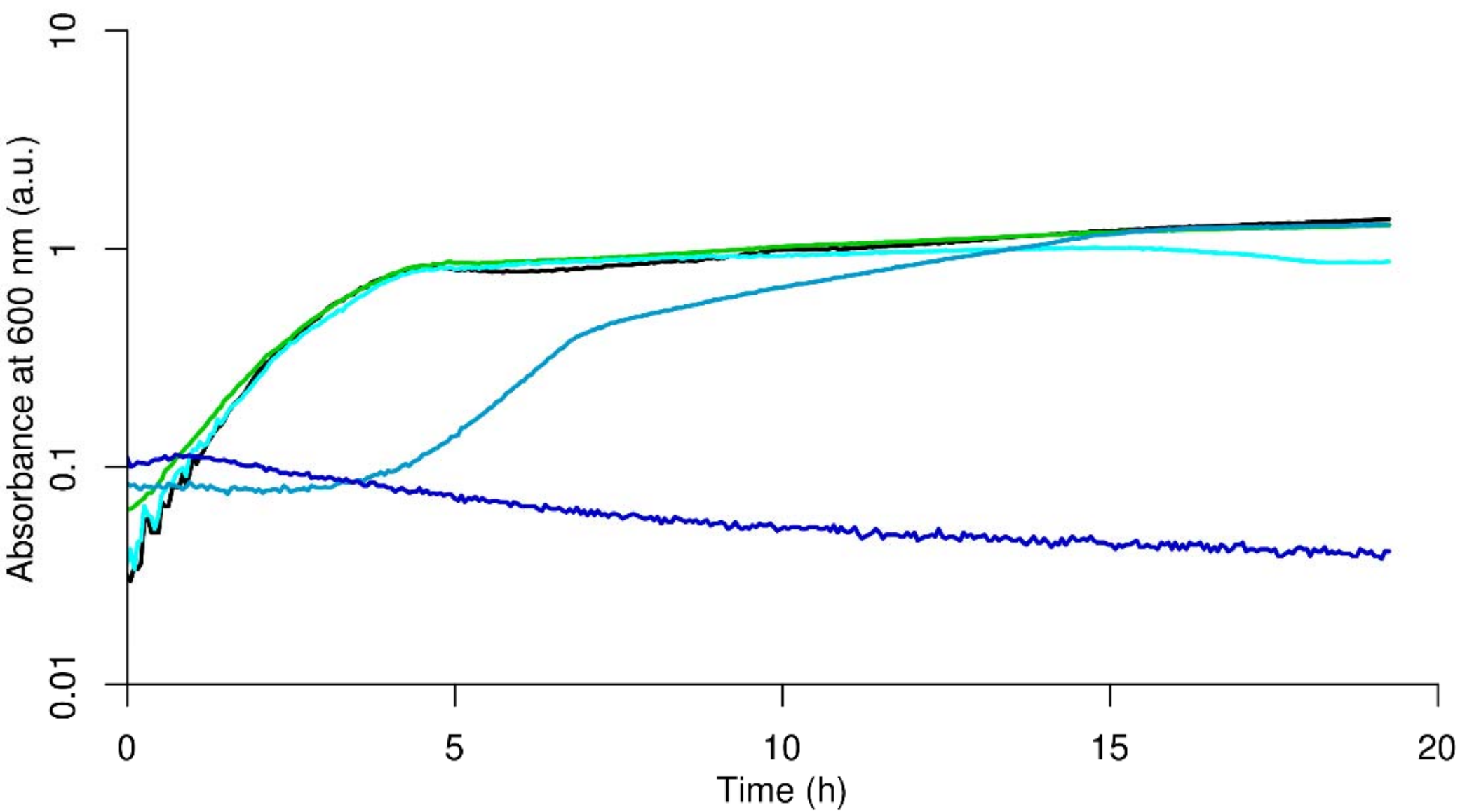

Supplement: FIG S1 [file mBio.01340-20-sf001.pdf]

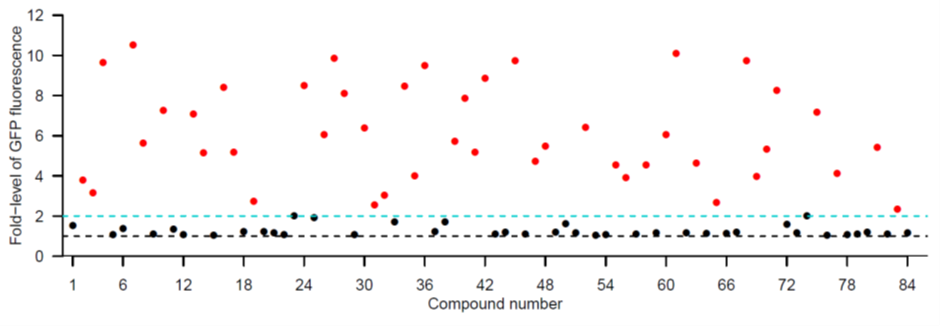

Supplement: FIG S2 [file mBio.01340-20-sf002.tif]

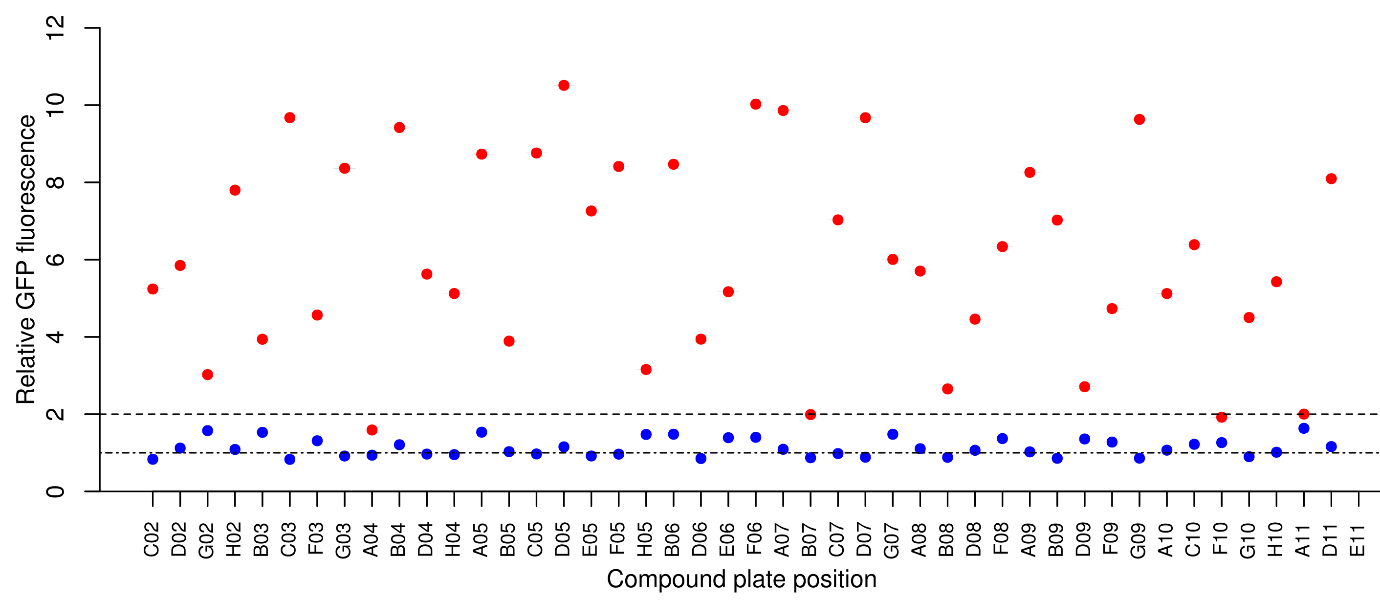

Supplement: FIG S3 [file mBio.01340-20-sf003.tif]

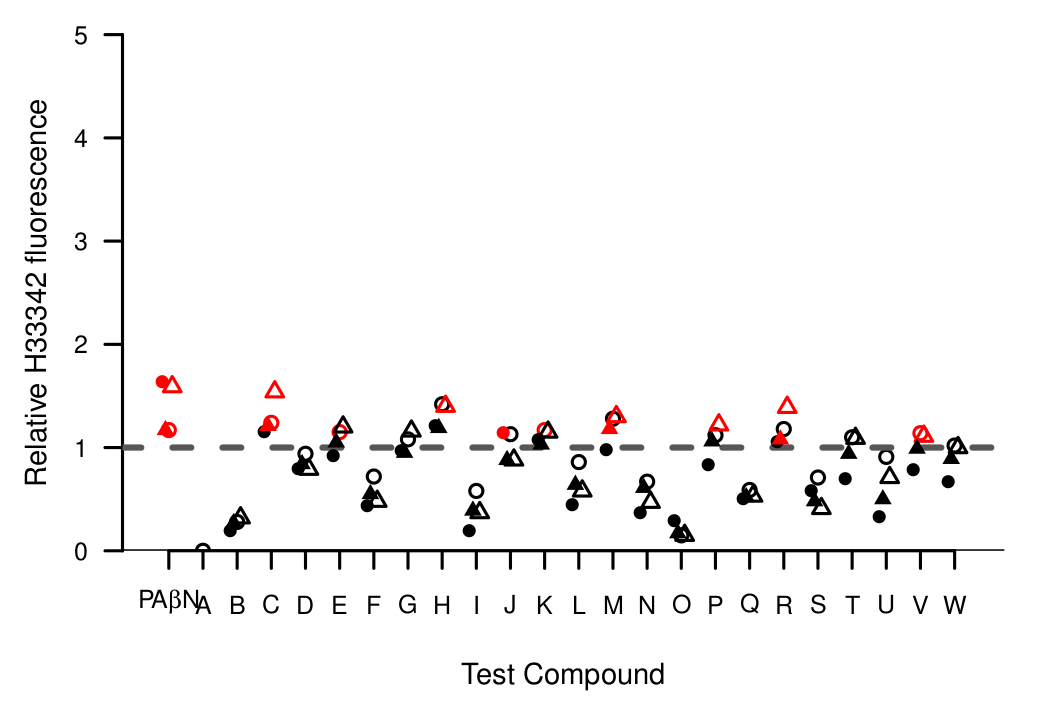

Supplement: FIG S4 [file mBio.01340-20-sf004.tif]

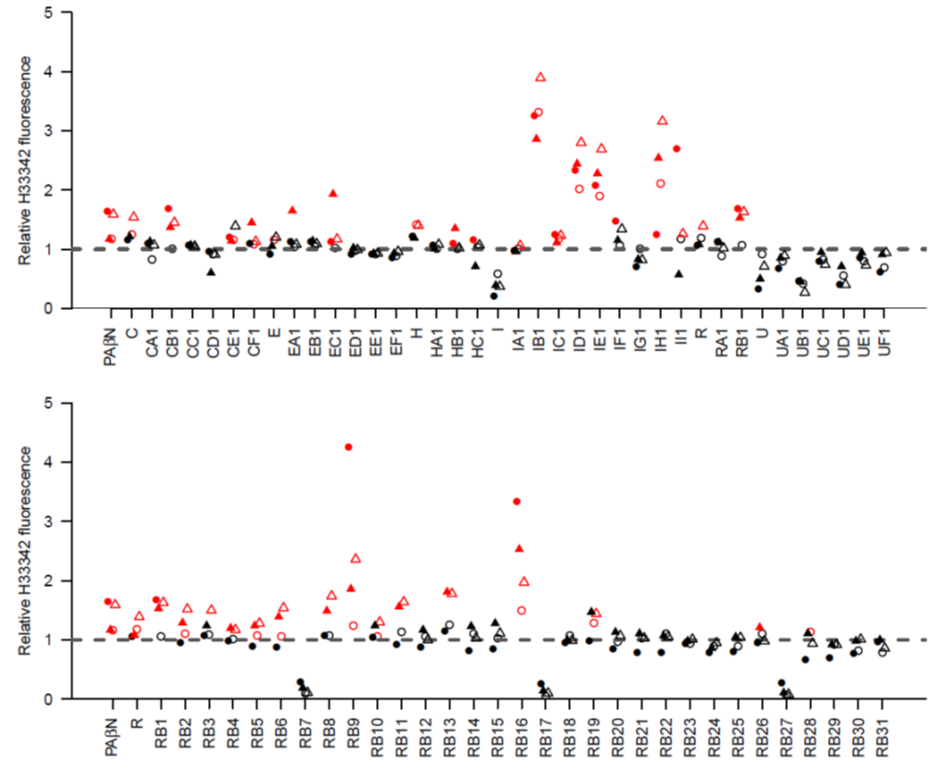

Supplement: FIG S5 [file mBio.01340-20-sf005.tif]
