# Supplementary material for: New Multidrug Efflux Inhibitors for Gram-Negative Bacteria
Source: mBio. 2020 Jul 14;11(4):e01340-20. doi: 10.1128/mBio.01340-20 (PMC7360932; doi:10.1128/mBio.01340-20)
Supplement: TABLE S1 [file mBio.01340-20-st001.docx]

**Supplementary Table 1. The 51 compounds from the Prestwick Chemical Library that caused ≥1.5-fold GFP fluorescence relative to the strain grown in MOPS minimal medium alone**

| **Compound** | **Fluorescence Fold Change** | **p-value** | **Compound Type** |
| --- | --- | --- | --- |
| Merbromin | 13.43 ± 1.44 | 0.052 | Topical antiseptic |
| Chloramphenicol | 5.74 ± 1.49 | 0.139 | Bacterial protein synthesis inhibitor |
| Florfenicol | 4.93 ± 1.67 | 0.185 | Bacterial protein synthesis inhibitor |
| Bepridil hydrochloride | 4.57 ± 1.54 | 0.188 | Anti-anginal |
| Clofazimine | 4.19 ± 1.7 | 0.230 | DNA-binding anti-(myco)bacterial |
| *Catharanthine* | 3.13 ± 0.01 | 1.1 x10^-8^ | Anti-cancer |
| Dipyridamole | 2.93 ± 0.73 | 0.166 | Anti-hypertensive |
| Rifapentine | 2.8 ± 0.12 | 0.025 | Bacterial RNA polymerase inhibitor |
| Prenylamine lactate | 2.7 ± 0.7 | 0.181 | Anti-hypertensive |
| Meclocycline sulfosalicylate | 2.32 ± 0.13 | 0.037 | Bacterial protein synthesis inhibitor |
| Chlortetracycline | 2.32 ± 0.64 | 0.209 | Bacterial protein synthesis inhibitor |
| Tetracycline | 2.23 ± 0.62 | 0.220 | Bacterial protein synthesis inhibitor |
| Rifampicin | 2.13 ± 0.29 | 0.112 | Bacterial RNA polymerase inhibitor |
| *Miconazole* | 2.11 ± 0.71 | 0.269 | Anti-fungal |
| Niridazole | 2.1 ± 0.06 | 0.009 | Anti-parasitic (trematodes) |
| Diphenylpyraline hydrochloride | 2.07 ± 0.07 | 0.017 | Anticholinergic Antihistamine |
| Zidovudine, AZT | 2.01 ± 0.34 | 0.148 | Antiretroviral |
| Thiamphenicol | 1.94 ± 0.11 | 0.050 | Bacterial protein synthesis inhibitor |
| Nisoxetine hydrochloride | 1.91 ± 0.01 | 1.4 x10^-12^ | Antipsychotic |
| *Thioridazine hydrochloride* | 1.88 ± 0.08 | 0.039 | Antipsychotic |
| Demeclocycline hydrochloride | 1.87 ± 0.39 | 0.194 | Bacterial protein synthesis inhibitor |
| Oxytetracycline dihydrate | 1.82 ± 0.13 | 0.069 | Bacterial protein synthesis inhibitor |
| Methiothepin maleate | 1.75 ± 0.23 | 0.204 | Antipsychotic |
| Tosufloxacin hydrochloride | 1.75 ± 0.32 | 0.186 | Bacterial topoisomerase inhibitor |
| Mebhydroline 1,5- naphtalenedisulfonate | 1.75 ± 0.36 | 0.132 | Anti-histamine |
| Perhexiline maleate | 1.74 ± 0.09 | 0.161 | Anti-anginal |
| *Mefloquine hydrochloride* | 1.74 ± 0.27 | 0.129 | Anti-parasitic (malaria) |
| Topotecan | 1.74 ± 0.22 | 0.042 | Anti-cancer |
| Cefixime | 1.72 ± 0.46 | 0.268 | Bacterial cell wall synthesis inhibitor |
| Doxycycline hydrochloride | 1.68 ± 0.27 | 0.175 | Bacterial protein synthesis inhibitor |
| Methacycline hydrochloride | 1.67 ± 0.05 | 0.012 | Bacterial protein synthesis inhibitor |
| Isoconazole | 1.64 ± 0.21 | 0.142 | Anti-fungal |
| Amphotericin B | 1.63 ± 0.3 | 0.202 | Anti-fungal |
| Minocycline hydrochloride | 1.63 ± 0.03 | 0.006 | Bacterial protein synthesis inhibitor |
| Daunorubicin hydrochloride | 1.62 ± 0.24 | 0.167 | Anti-cancer |
| Methapyrilene hydrochloride | 1.61 ± 0.15 | 0.014 | Anticholinergic/antihistamine |
| Fendiline hydrochloride | 1.61 ± 0.05 | 0.102 | Anti-hypertensive |
| Cefotetan | 1.61 ± 0.14 | 0.102 | Bacterial cell wall synthesis inhibitor |
| Ciprofloxacin | 1.6 ± 0.31 | 0.219 | Bacterial topoisomerase inhibitor |
| Clorgyline hydrochloride | 1.59 ± 0.01 | 1.5 x10^-9^ | Antipyschotic |
| Auranofin | 1.58 ± 0.14 | 0.102 | Anti-rheumatic |
| Moxalactam disodium salt | 1.57 ± 0.54 | 0.373 | Bacterial cell wall synthesis inhibitor |
| Cefoxitin sodium salt | 1.56 ± 0.4 | 0.301 | Bacterial cell wall synthesis inhibitor |
| Dicyclomine hydrochloride | 1.55 ± 0.15 | 0.118 | Anticholinergic |
| Cefdinir | 1.54 ± 0.21 | 0.246 | Bacterial cell wall synthesis inhibitor |
| 5-fluorouracil | 1.54 ± 0.31 | 0.171 | Anti-cancer |
| Quinacrine dihydrochloride dihydrate | 1.54 ± 0.26 | 0.211 | Anti-cancer |
| Chlorprothixene hydrochloride | 1.53 ± 0.12 | 0.091 | Antipyschotic |
| Azithromycin | 1.51 ± 0.38 | 0.308 | Bacterial protein synthesis inhibitor |
| Primaquine diphosphate | 1.51 ± 0.05 | 0.014 | Anti-parasitic |
| Dequalinium dichloride | 1.5 ± 0.08 | 0.045 | Quaternary ammonium compound |

Fluorescence fold-change is given as mean ± standard deviation, n = 2. P-values were obtained from a Student’s T-test for each compound with the compound-free control from the same plates.
