# Supplementary material for: New Multidrug Efflux Inhibitors for Gram-Negative Bacteria
Source: mBio. 2020 Jul 14;11(4):e01340-20. doi: 10.1128/mBio.01340-20 (PMC7360932; doi:10.1128/mBio.01340-20)
Supplement: TABLE S2 [file mBio.01340-20-st002.docx]

**Supplementary Table 2. MICs (µg/ml) of antibiotics and ethidium bromide for *E. coli*, *A. baumannii* and *P. aeruginosa* strains at the corresponding concentration of putative efflux inhibitor (μM).**

| ***E. coli* BW25113 *marR::aph*** | | | | | | | | | | |  | |  | | |  | | |  | | |  |  | |  | | |  | | |  | | |  | | |  |
| --- | --- | --- | --- | --- | --- | --- | --- | --- | --- | --- | --- | --- | --- | --- | --- | --- | --- | --- | --- | --- | --- | --- | --- | --- | --- | --- | --- | --- | --- | --- | --- | --- | --- | --- | --- | --- | --- |
| **Afn** | | **Chl** | | **Nal** | | **Tet** | | **EtBr** | | |  | **Cfz** | | | **Chl** | | | **Nal** | | | **Tet** | | **EtBr** |  | | **Dcm** | | | **Chl** | | | **Nal** | | | **Tet** | | |
| 0 | | 4 | | 4 | | 1 | | 128 | | |  | 0 | | | 4 | | | 4 | | | 2 | | 128 |  | | 0 | | | 4 | | | 32 | | | 2 | | |
| 6.25 | | 4 | | 4 | | 1 | | 128 | | |  | 3.1 | | | 4 | | | 4 | | | 2 | | 128 |  | | 400 | | | 4 | | | 32 | | | 2 | | |
| 12.5 | | 4 | | 4 | | 1 | | 128 | | |  | 6 | | | 4 | | | 8 | | | 1 | | 256 |  | | 800 | | | 4 | | | 32 | | | 2 | | |
| 25 | | 4 | | 4 | | 1 | | 64 | | |  | 13 | | | 4 | | | 4 | | | 2 | | 128 |  | | 1600 | | | 4 | | | 32 | | | 2 | | |
| 50 | | 4 | | 2 | | 0.5 | | 64 | | |  | 25 | | | 4 | | | 8 | | | 1 | | 128 |  | | 3200 | | | 4 | | | 32 | | | 2 | | |
| 100 | | 0.0625 | | 0 | | 0 | | 16 | | |  | 50 | | | 4 | | | 4 | | | 2 | | 128 |  | | 6400 | | | 0 | | | 32 | | | 1 | | |
|  | |  | |  | |  | |  | | |  | |  | | |  | | |  | | |  |  | |  | |  | | |  | | |  | | |  |  |
| ***A. baumannii* AB211** | | | | | | | | | | |  | |  | | |  | | |  | | |  |  |  |  |  |  |  |  |  |  |  |  |  |  |  |  |
| **Cfz** | **Chl** | | **Nal** | | **Tet** | | **EtBr** | |  | **Dcm** | | | | **Chl** | | | **Nal** | | | **Tet** | | |  |  |  |  |  |  |  |  |  |  |  |  |  |  |  |
| 0 | 128 | | 1024 | | >2048 | | 128 | |  | 0 | | | | 256 | | | 512 | | | >2048 | | |  |  |  |  |  |  |  |  |  |  |  |  |  |  |  |
| 3.1 | 128 | | 512 | | 1024 | | 128 | |  | 400 | | | | 128 | | | 512 | | | >2048 | | |  |  |  |  |  |  |  |  |  |  |  |  |  |  |  |
| 6 | 64 | | 512 | | 1024 | | 128 | |  | 800 | | | | 128 | | | 512 | | | >2048 | | |  |  |  |  |  |  |  |  |  |  |  |  |  |  |  |
| 13 | 64 | | 512 | | 1024 | | 128 | |  | 1600 | | | | 128 | | | 512 | | | >2048 | | |  |  |  |  |  |  |  |  |  |  |  |  |  |  |  |
| 25 | 64 | | 512 | | 1024 | | 128 | |  | 3200 | | | | 128 | | | 512 | | | >2048 | | |  |  |  |  |  |  |  |  |  |  |  |  |  |  |  |
| 50 | 64 | | 512 | | 1024 | | 128 | |  | 6400 | | | | 64 | | | 256 | | | 256 | | |  |  |  |  |  |  |  |  |  |  |  |  |  |  |  |
|  | |  | |  | |  | |  | | |  |  | | |  | | |  | | |  | |  |  | |  | | |  | | |  | | |  | | |
| ***P. aeruginosa* G365** | | | | | | | | | | |  |  | | |  | | |  | | |  | |  |  | |  | | |  | | |  | | |  | | |
| **Afn** | | **Chl** | | **Nal** | | **Tet** | | **EtBr** | | |  | **Cfz** | | | **Chl** | | | **Nal** | | | **Tet** | | **EtBr** |  | | **Dcm** | | | **Chl** | | | **Nal** | | | **Tet** | | |
| 0 | | 128 | | 512 | | 64 | | 2048 | | |  | 0 | | | 256 | | | 1024 | | | 128 | | 2048 |  | | 0 | | | 128 | | | 512 | | | 128 | | |
| 6.25 | | 256 | | 512 | | 32 | | 1024 | | |  | 3.1 | | | 256 | | | 1024 | | | 64 | | 2048 |  | | 400 | | | 256 | | | 512 | | | 128 | | |
| 12.5 | | 256 | | 512 | | 32 | | 1024 | | |  | 6 | | | 256 | | | 1024 | | | 64 | | 2048 |  | | 800 | | | 256 | | | 512 | | | 128 | | |
| 25 | | 256 | | 512 | | 32 | | 1024 | | |  | 13 | | | 256 | | | 1024 | | | 64 | | 2048 |  | | 1600 | | | 256 | | | 512 | | | 128 | | |
| 50 | | 256 | | 1024 | | 32 | | 512 | | |  | 25 | | | 256 | | | 1024 | | | 64 | | 2048 |  | | 3200 | | | 256 | | | 512 | | | 64 | | |
| 100 | | 256 | | 1024 | | 16 | | 128 | | |  | 50 | | | 256 | | | 1024 | | | 64 | | 2048 |  | | 6400 | | | 256 | | | 512 | | | 64 | | |

Chl, chloramphenicol; Nal, nalidixic acid; Tet, tetracycline; EtBr, ethidium bromide; Afn, auranofin; Cfz, clofazimine; Dcm, dicyclomine hydrochloride. **Bold font** indicates synergy, as determined by an FIC index <0.5.
