# Supplementary material for: New Multidrug Efflux Inhibitors for Gram-Negative Bacteria
Source: mBio. 2020 Jul 14;11(4):e01340-20. doi: 10.1128/mBio.01340-20 (PMC7360932; doi:10.1128/mBio.01340-20)
Supplement: TABLE S3 [file mBio.01340-20-st003.docx]

**Supplementary Table 3. MICs (µg/ml) of antibiotics and ethidium bromide for *E. coli*, *A. baumannii* and *P. aeruginosa* strains at the corresponding concentration of putative efflux inhibitor RB1 (μM).**

| ***S*. Typhimurium SL1344 *ramR::aph*** | | | | |  | ***E. coli* BW25113 *marR::aph*** | | | | |  |
| --- | --- | --- | --- | --- | --- | --- | --- | --- | --- | --- | --- |
| **RB1** | **Chl** | **Nal** | **Tet** | **EtBr** |  | **RB1** | **Chl** | **Nal** | **Tet** | **EtBr** |  |
| 0 | 8 | 16 | 4 | 512 |  | 0 | 8 | 4 | 2 | 256 |  |
| 3 | 8 | 16 | 4 | 512 |  | 3 | 8 | 4 | 2 | 256 | |
| 6 | 8 | 8 | 4 | 512 |  | 6 | 8 | 4 | 2 | 256 | |
| 12 | 8 | 8 | 4 | 512 |  | 12 | 4 | 4 | 2 | 256 | |
| 25 | 8 | 8 | 4 | 512 |  | 25 | 4 | 4 | 2 | 256 | |
| 50 | 8 | 8 | 4 | 512 |  | 50 | 4 | 4 | 2 | 128 | |
| 100 | 4 | **4** | 4 | 512 |  | 100 | 4 | 2 | 2 | 128 | |
| 200 | 4 | 2 | 2 | 256 |  | 200 | 2 | - | 1 | - |  |
|  |  |  |  |  |  |  |  |  |  |  | |
|  |  |  |  |  |  |  |  |  |  |  | |
| ***A. baumannii* AB211** | | | | |  | ***P. aeruginosa* G365** | | | | | |
| **RB1** | **Chl** | **Nal** | **Tet** | **EtBr** |  | **RB1** | **Chl** | **Nal** | **Tet** | **EtBr** | |
| 0 | 256 | 1024 | 1024 | 256 |  | 0 | 256 | 1024 | 64 | 2048 |  |
| 3 | 256 | 1024 | 1024 | 256 |  | 3 | 256 | 1024 | 64 | 2048 | |
| 6 | 256 | 1024 | 1024 | 256 |  | 6 | 256 | 1024 | 64 | 2048 | |
| 12 | 128 | 1024 | 512 | 256 |  | 12 | 256 | 1024 | 128 | 2048 | |
| 25 | **64** | 512 | 512 | 128 |  | 25 | 256 | 1024 | 128 | 2048 | |
| 50 | 32 | 256 | 128 | 32 |  | 50 | 256 | 1024 | 128 | 2048 | |
| 100 | - | - | - | - |  | 100 | 256 | 1024 | 128 | 2048 |  |
| 200 | - | - | - | - |  | 200 | 256 | 1024 | 64 | >2048 |  |

Chl, chloramphenicol; Nal, nalidixic acid; Tet, tetracycline; EtBr, ethidium bromide; Afn, auranofin; Cfz, clofazimine; Dcm, dicyclomine hydrochloride. **Bold font** indicates synergy, as determined by an FIC index <0.5.
